# The Global Challenge of Antimicrobial Resistance: Insights from Economic Analysis

**DOI:** 10.3390/ijerph7083141

**Published:** 2010-08-09

**Authors:** Karen Eggleston, Ruifang Zhang, Richard J. Zeckhauser

**Affiliations:** 1 Shorenstein Asia-Pacific Research Center, Stanford University, 616 Serra Road, Encina Hall, Stanford, CA 94305, USA; 2 Global Investment Research, Goldman Sachs International, Peterborough Court, 133 Fleet Street, London EC4A 2BB, UK; E-Mail: ruifang.zhang@gs.com; 3 John F. Kennedy School of Government, Harvard University, 79 John F. Kennedy Street, Cambridge, MA 02138, USA; E-Mail: richard_zeckhauser@harvard.edu

**Keywords:** antimicrobial resistance, economic analysis, drug resistance, behavioral economics

## Abstract

The prevalence of antimicrobial resistance (AR) limits the therapeutic options for treatment of infections, and increases the social benefit from disease prevention. Like an environmental resource, antimicrobials require stewardship. The effectiveness of an antimicrobial agent is a global public good. We argue for greater use of economic analysis as an input to policy discussion about AR, including for understanding the incentives underlying health behaviors that spawn AR, and to supplement other methods of tracing the evolution of AR internationally. We also discuss integrating antimicrobial stewardship into global health governance.

## 1. Introduction

The prevalence of drug resistance limits the therapeutic options for treatment of infections, and contributes to the global specter of a “post-antimicrobial era” in which some of the most effective tools in the physician’s armamentarium—including antibiotics, anti-tuberculosis and anti-malarial drugs—lose their effectiveness [1–8].

The objective of this paper is to illustrate how economic analysis can be a useful input to policy discussion about AR in at least three areas: understanding the incentives for health behaviors that contribute to resistance; analyzing the evolution of international resistance patterns; and contributing toward effective international governance of AR. We first discuss economic incentives surrounding health behaviors that lead to development of drug resistance. Next, to illustrate the potential for economic analysis to contribute to understanding the global evolution of resistance, we report a preliminary study of the correlation between resistance patterns across three selected countries. We conclude with a short discussion of proposals for confronting the antimicrobial resistance challenge and a summary of our arguments.

## 2. Behavior and the Economics of Antimicrobial Resistance

Antimicrobial agents are drugs that suppress the growth and replication of microorganisms such as bacteria, fungi and viruses. Medical treatment with antimicrobials imposes a selection pressure on microbes. For example, antibiotics kill weaker bacteria and select the stronger bacteria as survivors. Microorganisms resistant to one particular antimicrobial can develop resistance against others with similar pharmacological methods, giving rise to multi-drug resistance [9–10]. Because resistance confers an advantage in a world of widespread antimicrobial use, surviving pathogens pass on the genetic codes for resistance to their posterity.

The applicability of economics to the problem of antimicrobial resistance extends far beyond the obvious and important point that treatment costs are far higher for infections caused by antimicrobial-resistant organisms than for infections due to antimicrobial-susceptible organisms [11]. The behavior of numerous diverse individual consumers, patients, health care providers, and distributors contribute to antimicrobial resistance, involving many issues long considered central to economic analysis.

Antimicrobial use often benefits people other than the patient—creates a positive externality—by helping to control the spread of infection. However, antibiotic use also selects for resistant strains, contributing to the problem of antimicrobial resistance. This latter negative externality arises in part because each user does not bear the negative repercussions of future resistance when deciding how much of a drug to use, thus tending to use more of the drug than would be socially desirable. One example of such “over-use” or “misuse” would be taking an antibiotic to treat a viral infection. Widespread use of antibiotics to promote livestock growth also contributes to selective pressure on microbes [12].

Under-use also promulgates resistance. Poor patient adherence to antimicrobial regimens increases selective pressure. Lack of ability to pay and shortages of antimicrobials “promote underdosing, the substitution of available but unsuitable drugs, procurement from inappropriate sources, and drug counterfeiting. Therefore, to avoid compromising therapy and promoting resistance, antimicrobials may need to be made more (rather than less) available in certain instances, provided their availability is intelligently controlled and effective therapeutic doses are adhered to” [13, p. 572]. Patients contribute to their own and their children’s vulnerability to expensive-to-treat or even incurable infectious disease by taking drugs in too small a dosage or for too short a time period to be effective—except in contributing to resistance.

Under-use often co-exists with overuse of low-price, first-line drugs. Both breed resistance. Analyzing the underlying incentives driving under- and over-use can be important for understanding how policies will impact AR. Consider, for example, the likely effects of recent insurance expansions towards universal coverage in the U.S. and China. Increased access to care, including medications, can exacerbate AR when overuse is already a problem because of high levels of use (in the U.S.) or strong incentives for over-prescribing (in China). By contrast, among the poorest in China and in low-income countries, expanded access to care could potentially slow the pace of AR by enabling patients to access a full course of treatment or use a most costly but more effective and less resistance-prone medication.

For example, poor malaria patients may choose an inexpensive single drug over a more expensive combination therapy, even though the latter is both more likely to cure them as well as to preserve treatment effectiveness for others. To confront this dilemma, a report from the U.S. Institute of Medicine recommends large global subsidies for antimalarial drugs known as artimisinin combination therapies [14]. Laxminarayan *et al.* [15, p. 325] find that “even a partial subsidy could delay the emergence of resistance and that a delay in implementing a subsidy for artemisinin-based combination treatments could facilitate the emergence of resistance and lower the economic value of combination treatments.” In this way, economic incentives can be harnessed to encourage appropriate use and reduce AR.

Economic evaluations of global health initiatives should also take account of AR. Development of resistance, an unavoidable side effect of even prudent antibiotic use, accelerates when antimicrobials are used inappropriately. Because of this negative externality from antimicrobial treatment, the social benefit from disease prevention is higher than it otherwise would be. Unfortunately this extra benefit from prevention is not routinely incorporated into the evidence base used to inform global population health policies.

Another potentially important contributor to the resistance problem that we have not seen highlighted elsewhere is the well-documented human desire for instant gratification [16–17]. Patients and their loved ones want immediate cure, even if in the long run they would prefer everyone used drugs prudently. Overuse of antibiotics in high-income countries probably illustrates this best: parents may desire “instant gratification” to relieve a child’s ear infection, so the child can return to school and the parent to work or other pressing activities. (Ironically, most parents also want to teach children to think longer-term by, for example, investing in education and eschewing instant gratification from over-eating or substance abuse.)

Moreover, widespread use of antibiotics in agriculture, food animals, and aquaculture also contributes to AR, with negative consequences for preserving antimicrobial effectiveness in human medicine [18–20]. The importance of antimicrobial effectiveness suggests that further research into the behaviors undermining it merits priority.

## 3. The Global Evolution of Antimicrobial Resistance

**Figure 1.**
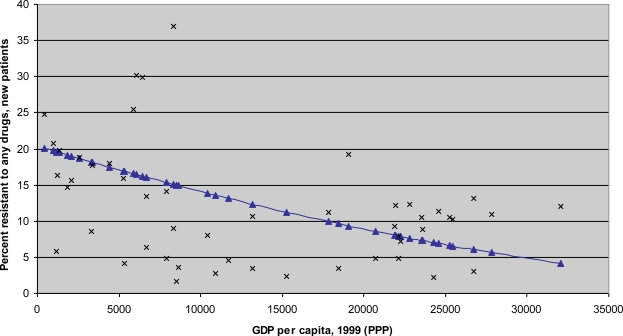
The relationship between GDP per capita and resistance to any anti-Tuberculosis drug. Source: Authors’ calculations based on resistance data from [22]. The resistance data is for 1999 or latest available year at the time of that publication. The curve in blue results from weighting each country’s TB resistance rate by that country’s TB population. The TB population in each country is calculated by multiplying its TB prevalence rate by its total population. For two large countries with different reported resistance rates across provinces, China and Russia, the national resistance rates are calculated as a weighted average of the reported provincial resistance rates, where the weights represent the TB population of the relevant province(s). Data on Gross Domestic Product (GDP) per capita for 1999 comes from the World Bank’s World Development Indicators database, available through http://www.worldbank.org/data/. GDP per capita is expressed in internationally comparable dollars using purchasing power parity (PPP).

Numerous scholars have chronicled the seemingly inexorable increase in antimicrobial resistance and its costs, including increased treatment spending, greater morbidity, and higher mortality. In a recent review of antimicrobial resistance in developing countries, Okeke *et al.* [12, p.481] find that “the general picture is one of accelerating rates of resistance spurred by antimicrobial misuse and shortfalls in infection control and public health. Reservoirs for resistance may be present in healthy human and animal populations.” In particular, the accumulating evidence base suggests that “the prevalence of resistance in seminal developing country pathogens is high and rising” [13, p.568]. For example, Figure 1 illustrates the high resistance rates to anti-Tuberculosis drugs in areas with low per capita resources. (By contrast, high-income countries like the US have among the world’s highest rates of resistance to second- and third-line drugs, such as vancomycin among antibiotics [21].)

Of course, the problem of AR is exceedingly complex; we are not suggesting that merely examining correlations between GDP and resistance will by itself contribute to an effective policy response. Many environmental, behavioral, financial and institutional factors shape the problem. Our point is simply that further research should seek to uncover how economic analysis can complement other disciplines in contributing to a better understanding of the complicated forces driving AR.

For example, the literature to date provides little evidence on whether the pace of integration of global commodity markets and flows of people affect countries’ patterns of antimicrobial resistance. Yet with globalization trends accelerating, economic analysis of international resistance patterns could be a useful input to policy discussion.

Clearly countries’ burdens of disease and healthcare practices shape the constellation of antimicrobial use and selection pressure. Correlation of resistance patterns across countries could arise for a number of reasons. Ease of international travel might lead to convergence of resistance patterns among countries that deal more with each other. However, some drugs—such as first-generation and cheaper antimicrobials—are more likely to see widespread use across countries of widely varying resources regardless of global openness, and some “bug-drug” pairs are simply more prone to develop resistance.

To begin to examine these issues, we constructed coefficients of resistance correlation among three countries for which we had some comparable data: China, the U.S. and Kuwait. China and the U.S. are both large, diverse countries that are major consumers of antibiotics. Kuwait is included to examine patterns for a far smaller economy that is geographically distant from the U.S. and China [21]. We ranked resistance rates for 24 “bug-drug” pairs and defined perfect correlation as each bug-drug pair displaying the same resistance rank. Perfect negative correlation would exist if the ranks in two countries go in precisely the opposite order.

More specifically, we compute a Spearman’s correlation coefficient statistic as follows:

$$C=1-\frac{6\sum_{i=1}^{24}{\left({\text{rk}}_{i,a}-{\text{rk}}_{i,b}\right)}^{2}}{24(24^{2}-1)}$$

where i = pair i of the 24 pairs and *rk_a_* and *rk_b_* represent ranks of bug-drug pair i in countries a and b, respectively. The statistic by definition is bounded between −1 and 1, where −1 means perfect disagreement while 1 means perfect agreement. Thus the bigger the statistic, the more correlated the two countries’ resistance patterns are.

We find that resistance rates in China are much more strongly correlated with those in Kuwait than those in the U.S. Specifically, the Spearman’s correlation coefficient as defined above is low (0.18) and not statistically significant (t = 0.85) for the U.S. and China; of moderate magnitude and significance (0.46, t = 2.43) for the U.S. and Kuwait; and strong and highly significant (0.60, t = 3.52) for China and Kuwait [21]. These findings appear to indicate that resistance in a country is determined primarily by country-specific factors associated with economic development, such as strictness of practices for prescribing drugs. It would be interesting to study such patterns of correlation across a broader set of countries, as well as how the patterns evolve over time.

Numerous scientific techniques make tracing patterns of antimicrobial resistance increasingly possible and less costly. Drug susceptibility testing is critical for appropriate clinical treatment, and that data can feed into surveillance efforts. Identification of specific antibiotic resistance genes is becoming increasingly feasible in some settings as well [23]. To these methods we suggest adding social science research to examine the correlations between patterns of antimicrobial resistance and socioeconomic factors such as social determinants of health-seeking behavior, healthcare provider incentives, and measures of economic integration across regions. Metrics of drug resistance correlation await further refinement. We hope that others will build upon these methods for examining the global evolution of antimicrobial resistance.

## 4. Integrating Antimicrobial Stewardship into Global Health Governance

Like an environmental resource, antimicrobials require stewardship. The effectiveness of an antimicrobial agent is a global public good. (Rudholm [24] provides a theoretical analysis of this issue). No patent protects a drug from overuse or inappropriate use that leads to resistance. Because antimicrobial effectiveness is a global public good, international cooperation to curb antimicrobial resistance has elements of a classic “prisoners’ dilemma”: individuals and countries fail to coordinate on prudent use, because high temptations to deviate (or free ride on others’ prudence) allow descent to the undesired (high-resistance) equilibrium.

Robust development of new antimicrobials could postpone the return of a ‘pre-antibiotic era’ and reduce the aggregate resistance burden. The initial development and use of new drugs, and the associated decrease in burden from resistant diseases, is most likely in high-income countries. To what extent these technological breakthroughs diffuse internationally will determine whether they are a force for convergence or divergence of standards of living, in an age already characterized by extreme inequalities in life opportunities [25]. Unfortunately, for many important pathogens, few new antimicrobial agents have been developed recently. But increased awareness and appropriate economic incentives could stimulate renewed innovation both in drugs and diagnostics.

One cause for optimism is the recent emergence of numerous international organizations and cooperation schemes addressing different facets of the antimicrobial resistance problem. Examples include the Alliance for the Prudent Use of Antibiotics, Resources for the Future, ReAct—Action on Antibiotic Resistance, the International Networks for the Study and Prevention of Emerging Antimicrobial Resistance, the International Network for Rational Use of Drugs, The Antimicrobial Resistance Prevention Initiative, the Medicines for Malaria Venture, the Global Alliance for TB Drug Development, and various initiatives of the World Health Organization (such as its Global Gonococcal Antimicrobial Susceptibility Program, the WHO/International Union against Tuberculosis and Lung Disease Global Project, and WHONET shareware to standardize analysis of susceptibility testing results [13]).

Strategies with promise for containing antimicrobial resistance include education for prescribers, distributors, and patients; national promulgation of combination therapies or cycling of drugs; programs such as the Integrated Management of Childhood Illnesses and Directly Observed Shortcourse Therapy for Tuberculosis; regulation of drug quality; and policies to address “cultures of antimicrobial abuse” [13, p.568]. An effective and cost-effective response will also need to promote global surveillance; evaluate market-based incentives such prudent drug use measures in provider “pay-for-performance” schemes (since provider payment methods can substantially affect prescribing and resistance trends, as South Korea’s experience reveals [26]); and experiment with essential drug lists, prescribing guidelines, and new ways of making and marketing drugs to discourage inappropriate self-medication. Useful ideas might come from creative yet rigorous economic analysis, such as applying behavioral economics to design interventions to promote rational drug use, or incorporating the benefits of reduced antimicrobial resistance in all economic analyses of prevention. A global perspective will be imperative in encouraging innovation in drugs and diagnostics while balancing the need for increasing access and affordability (since, as noted above, under-use as well as over-use contributes to AR). Policymakers should consider creative solutions such as tiered pricing alongside other mechanisms for balancing these competing needs. Economists should also contribute to analysis of trends in specific countries, and how health system reforms do or do not contribute to better antimicrobial stewardship. For example, as pointed out by Heddini *et al.* [27], the rate of antibiotic resistance in China is alarmingly high and growing rapidly; expansion of insurance may even exacerbate the problem. A glimmer of hope comes from the 2009 health reforms’ inclusion of policies to support more rational drug use.

## 5. Conclusions

It was not so long ago that prominent reports on environmental challenges failed to identify global climate change as a key threat, yet few other issues have greater prominence on the environmental agenda for the twenty-first century [28,29]. Drawing a parallel to antimicrobial resistance is natural. The challenge of antimicrobial resistance should move to the forefront of the global public health agenda in the decades to come.

We argue that in at least three important arenas, economic analysis can be a useful input for policy about AR: analyzing and shaping the incentives that drive health behaviors such as over-use and under-use that contribute to resistance; quantifying the evolution of international resistance patterns; and helping to understand how public-private partnerships can contribute to effective international governance of antimicrobial effectiveness as a global public good.

This paper has several limitations. We confine ourselves to a short discussion of some salient issues; a more detailed exploration awaits further research. Our illustration of a potential method for tracing evolution of resistance patterns includes only three countries and a limited number of “bug-drug” pairs for each country. No claim can be made of global representativeness or generalizability. Moreover, some of the economic concepts we apply may be less familiar to medical or environmental science experts. We hope the limitations of our analysis will prompt others to pursue further research to uncover how economic analysis can complement other disciplines in contributing to a better understanding of the complicated forces driving resistance.

In sum, many institutional, environmental, behavioral, and financial factors shape the global AR problem. Health economists and other social scientists studying health policy should play an important role in understanding the behaviors that underlie AR and how to design incentives to coordinate a global response.
